# Supplementary material for: Modulation of plant root growth by nitrogen source‐defined regulation of polar auxin transport
Source: EMBO J. 2021 Jan 5;40(3):e106862. doi: 10.15252/embj.2020106862 (PMC7849315; doi:10.15252/embj.2020106862)
Supplement: Supplementary file 1 — Appendix [file EMBJ-40-e106862-s001.pdf]

APPENDIX Ötvös et al.

Table of contents:

Appendix Fig. S1 | Additional data supporting the distinct division patterns in Col-0 roots on media supplemented with different nitrogen source.

Appendix Fig. S2 | Supporting data for auxin activity and distribution in roots transferred to different nitrogen sources.

Appendix Fig. S3 | Additional data for demonstrating the distinct patterns of auxin distribution in Col-0 and *eir1-4* roots.

Appendix Fig. S4 | Additional data for demonstrating root growth phenotypes and cell divisions in pin2 mutants.

Appendix Fig. S5 | PIN2 immunostaining in PIN2-GFP expressing roots.

Appendix Fig. S6 | Additional data supporting impact of PIN2S439 phospho-variants on root growth adaptation to differing source of nitrogen.

Appendix Fig. S7 | Model robustness and fitting to experimental measurements.

Appendix Fig. S8 | Model diagram.

Appendix Fig. S9 | Statistical reasoning.

Appendix Fig. S1

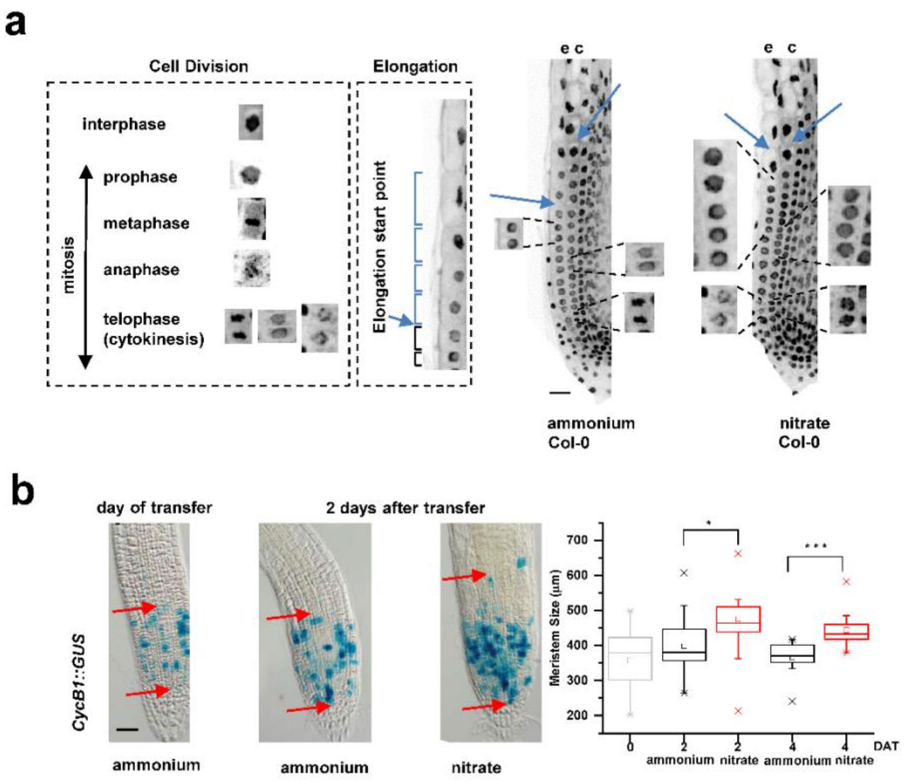

Appendix Fig. S1 | Additional data supporting the distinct division patterns in Col-0 roots on media supplemented with different nitrogen source.

a. On the left, schematic representation of the different cell division phases of DAPI stained Col-0 roots and an illustration of how the onset of cell elongation was marked. On the right, DAPI stained confocal microscopic images of Col-0 roots 12 HAT to ammonium or nitrate containing medium. Mitotic events are displayed along epidermis (e) and cortex (c). Blue arrows point to the first elongating cells. Scale bar=50 µm. **b.** Bright field microscopic images of *GUS* expressing roots driven by *CycB1* promoter. Blue spots mark *CycB1* promoter activity. Red arrows point to the beginning and to the end of GUS expressing area (meristem size). Scale bar=100µm. Box plot chart represents the meristem size (µm) of *CycB1::GUS* expressing roots on the day of transfer (0) and 2 and 4 DAT to ammonium or nitrate. Differences of the means were calculated with a t-test (p value *<0.05, ***<0.001). At least 14 roots were analyzed per time point per treatment.

Appendix Fig. S2

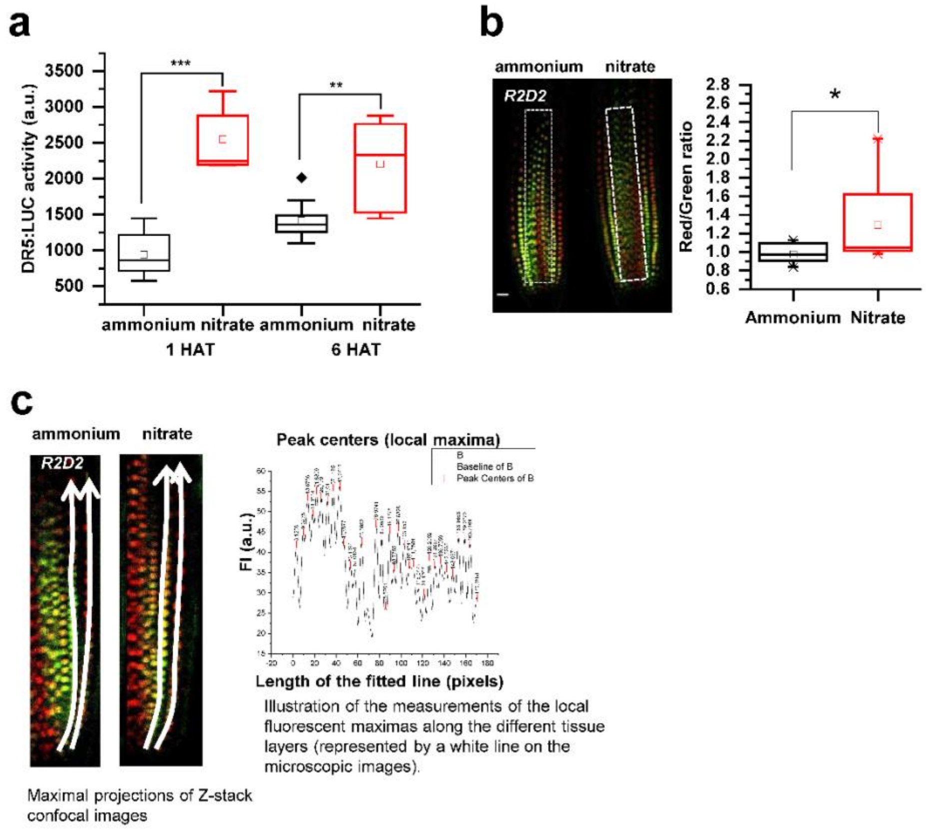

Appendix Fig. S2 | Supporting data for auxin activity and distribution in roots transferred to different nitrogen sources.

a. *DR5* activity in *Arabidopsis* roots. Box plots represents luciferase activities in *DR5::LUC* expressing roots 1 and 6 HAT. 40 roots were collected per treatment per time points. Experiment was repeated 3 times. Statistical differences were calculated with a t-test (p values **<0.01, ***<0.001) **b.** Expression profile of the auxin-input reporter *R2D2* in the stele (labeled with “white box”) of roots 12 HAT to ammonium or nitrate containing media. Box plots represent the quantification of the red (auxin-independent) vs green (auxin dependent) fluorescent signal ratio in the stele. At least 13 roots were analyzed and statistical difference was calculated with a t-test (p value <0.05). **c.** Illustrations for R2D2 quantification along epidermal and cortical cell files. For details see the “Quantification of R2D2, DII-VENUS and mDII-VENUS fluorescence signal” in the “Methods” section.

Appendix Fig. S3

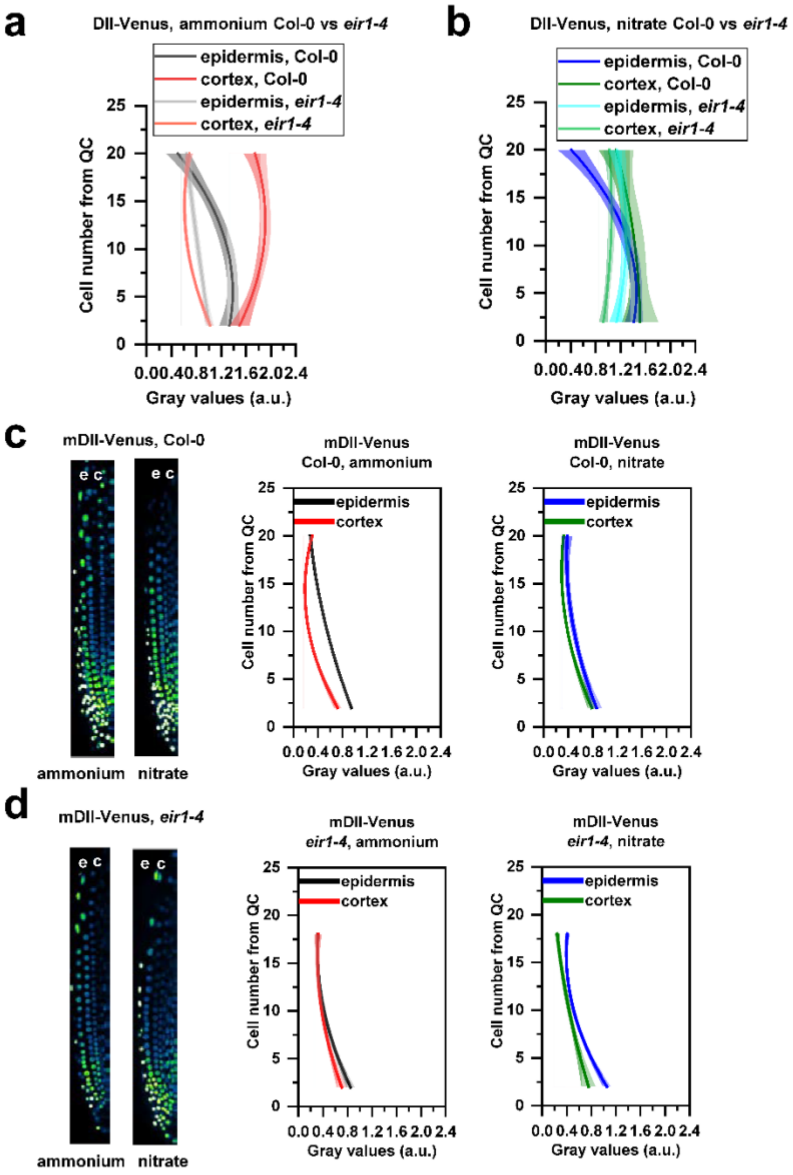

Appendix Fig. S3 | Additional data for demonstrating the distinct patterns of auxin distribution in Col-0 and *eir1-4* roots.

a and **b.** Comparison of DII-Venus fluorescent signal in Col-0 and *eir1-4* lines on ammonium (**a**) and on nitrate (**b**) transferred roots. Graphs denote normalized relative auxin levels at the respective positions. Lines represent polynomial regression fit with 95% confidence band. Data are derived from measurements of n=8 (ammonium) and n=10 (nitrate) roots of Col-0 and n=10 roots of *eir1-4* per condition. **c** and **d.** Maximum intensity Z-stack projection images of 5 DAG old Col-0 (**c**) and *eir1-4* mutant (**d**) roots expressing the non-auxin degradable mDII-Venus reporter grown on ammonium and nitrate supplemented media 12 HAT. “e” and “c” marks epidermis and cortex, respectively. Scale bar=50 µm. Graphs denote grey values (arbitrary units - a.u.) at the respective positions. Lines represent polynomial regression fit with 95%

confidence band. Data are derived from measurements of at least 5 roots per genotype per treatment.

Appendix Fig. S4

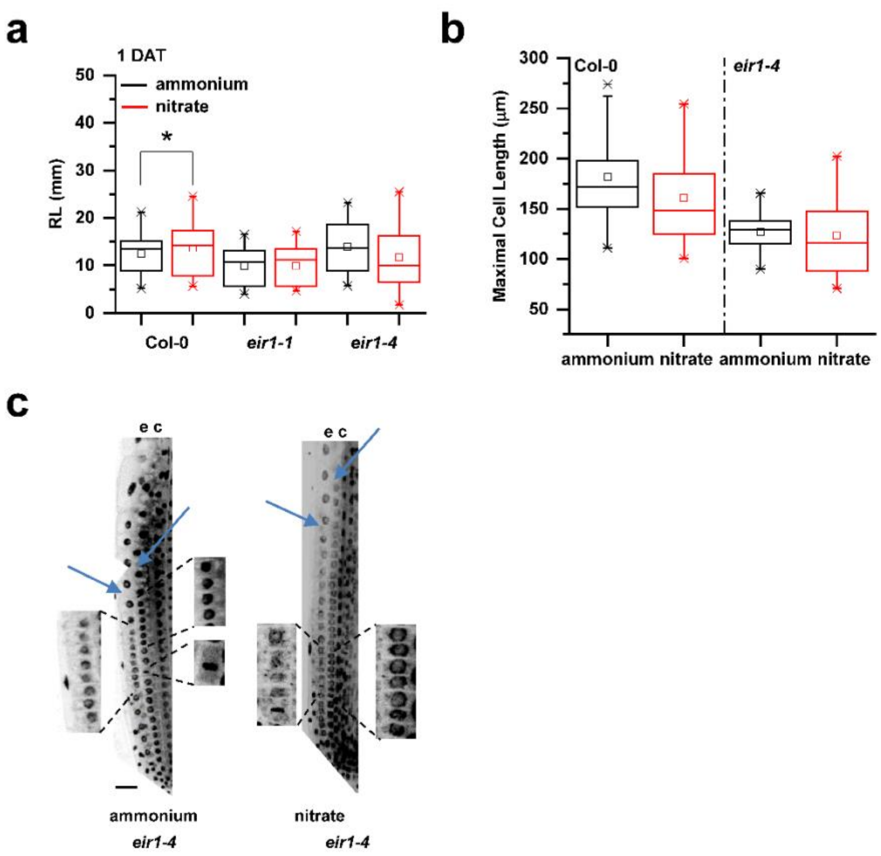

Appendix Fig. S4 | Additional data for demonstrating root growth phenotypes and cell divisions in *pin2* mutants.

a. Comparison of root length of *pin2* mutants (*eir1-1* and *eir1-4*) to Col-0 on ammonium (black) and nitrate (red) amended media, 1 DAT. At least 11 roots were analyzed per genotype per treatment and statistical difference was calculated with a t-test (p value <0.001) **b.** Box plots of the maximal cell length of Col-0 and *eir1-4* mutant roots 12 HAT to ammonium (black) or nitrate (red). 3-3 cells in at least 13 roots were analyzed per genotype per treatment. **c.** DAPI stained confocal microscopic images of *eir1-4* roots 12 HAT to ammonium or nitrate containing medium. Mitotic events are highlighted along epidermis (e) and cortex (c). Blue arrows point to the first elongating cells. Scale bar=50 µm.

Appendix Fig. S5

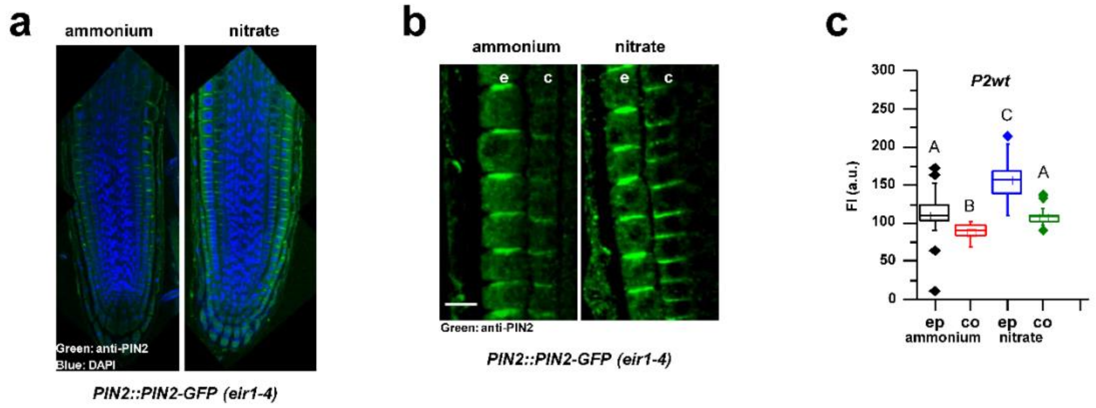

Appendix Fig. S5 | PIN2 immunostaining in *PIN2-GFP* expressing roots.

a. Confocal microscopic images show anti-PIN2 immunostained PIN2-GFP expressing root tips 12 HAT to ammonium or nitrate. Green and blue signal represents PIN2 and nuclear staining with DAPI, respectively. **b.** Higher magnification of anti-PIN2 immunostained *PIN2-GFP* expressing root in the transition zone. “e” and “c” denote epidermis and cortex, respectively. Scale bar=25 µm. **c.** Quantification of PIN2 fluorescent signal in the immunostained, ammonium or nitrate treated roots in epidermal (ep) and cortical (co) cell files. Statistical difference was evaluated with ANOVA at $p<0.05$. 10 roots were analyzed per treatment.

Appendix Fig. S6

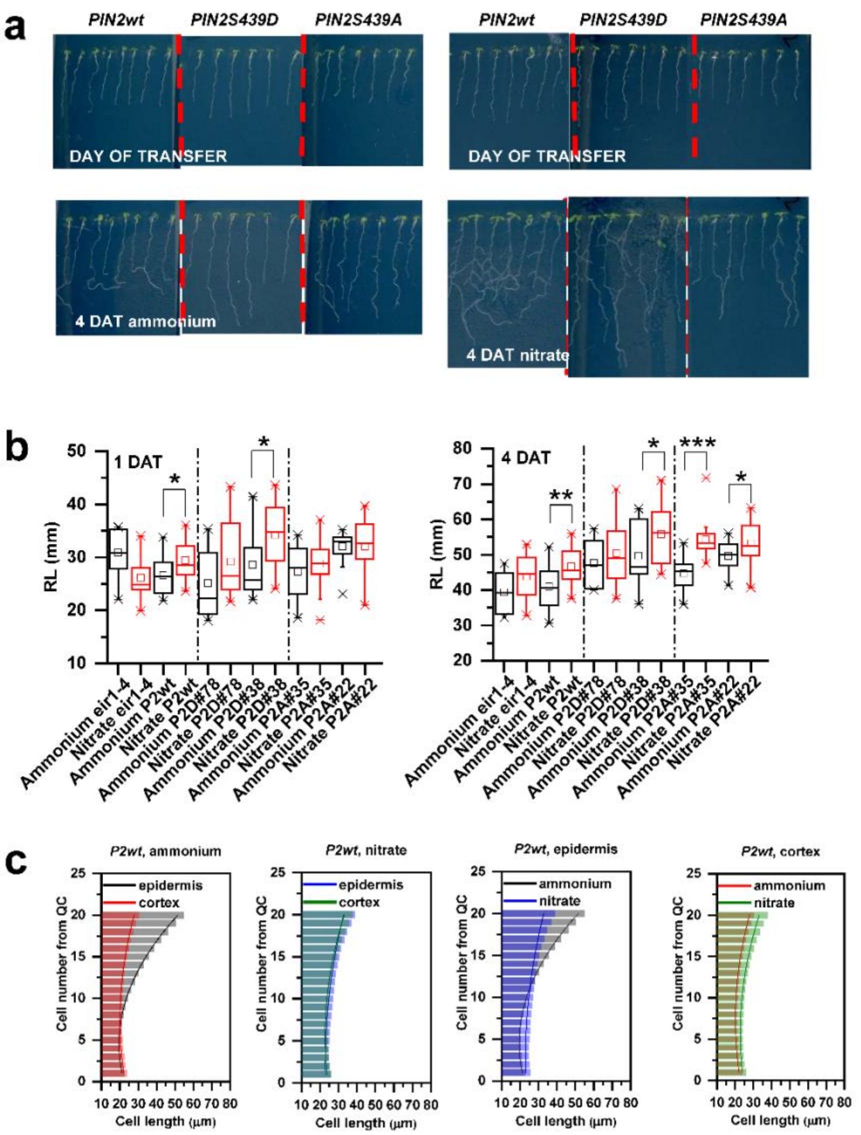

Appendix Fig. S6 | Additional data supporting impact of PIN2S439 phospho-variants on root growth adaptation to differing source of nitrogen.

a, b. Seedlings expressing *PIN2::PIN2-GFP* (*P2wt*), *PIN2::PIN2S439D-GFP* (*P2D*) and *PIN2::PIN2S439A-GFP (P2A)* in *eir1-4* background. Representative images of seedlings at the day of transfer and 4 DAT to ammonium or nitrate supplemented plates are shown (**a**). Quantification of root length (mm) in *eir1-4, PIN2wt (eir1-4)* and two independent *P2D (eir1-4)* (#78 and #38) and *P2A (eir1-4)* (#35 and #22) lines 1 and 4 DAT to ammonium or nitrate containing media. At least 7 roots per genotype per treatment were analyzed. Statistical difference was evaluated with a t-test (p values *<0.05, **<0.01 and ***<0.001). **c.** Comparison of cell length changes in epidermal and cortical cell files of *PIN2::PIN2-GFP*

(*P2wt*) expressing roots transferred to ammonium or nitrate. Column bars denote the geometric mean of cell length at the respective positions. Lines represent a polynomial regression fit. Data are derived from measurements of 20 roots per genotype per treatment.

Appendix Fig. S7

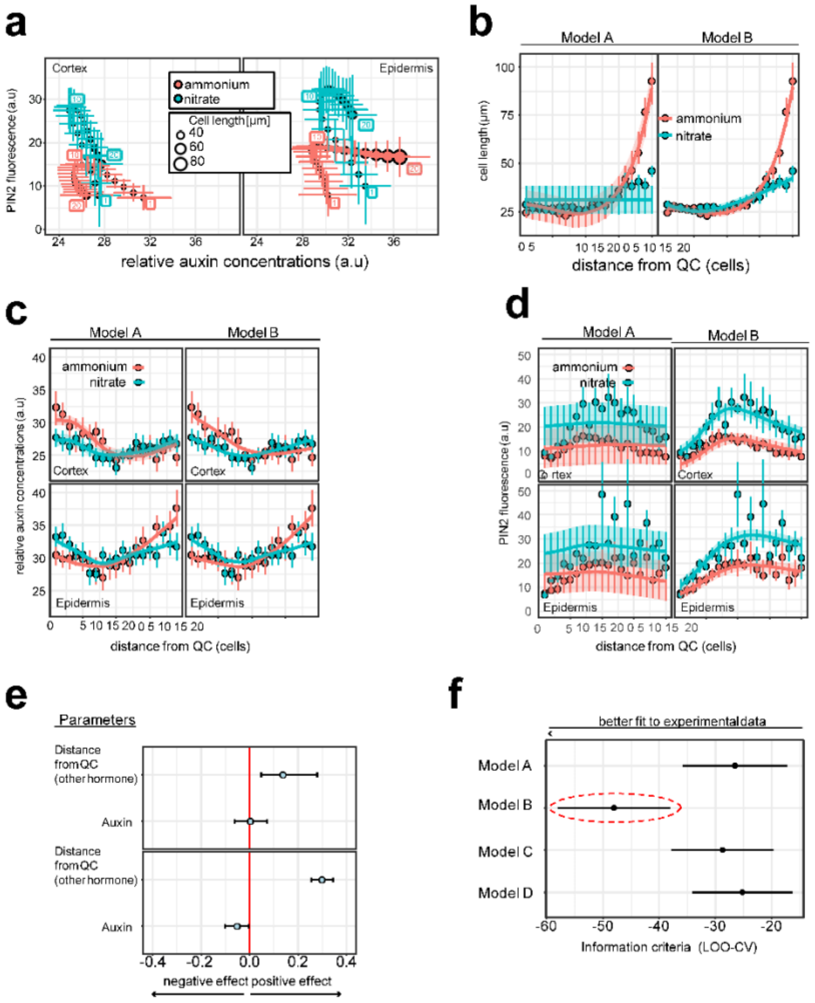

Appendix Fig. S7 | Model robustness and fitting to experimental measurements.

a. The experimentally-driven PIN2 levels in the function of relative auxin levels for cortex (left panel) and epidermis (right panel), respectively. Relative auxin levels were calculated as follows: log(G/(G+R)) / -0.025, where G and R represent DII-Venus (green) and mDII-Tomato (red) fluorescent signals, respectively. Cell length is denoted by dot size, and the distance of the cell from the QC is labelled with consecutive numbers. Vertical and horizontal lines represent the standard error of measurements. **b-d.** Validations in two regimes demonstrate that Model B faithfully recapitulates all experimental measurements. In these plots model predictions (thick lines and shaded areas for the posterior average and 95% confidence intervals, respectively) are plotted against experimental data (dots and vertical bars for data mean and standard deviations, respectively). e. Parameter estimations for Model B suggest antithetic cumulative effects of auxin level and distance from QC on PIN2 dynamics. **f.**

Predictive power of four models for auxin input scenario (A-D). The expected log probability density is used as information criterion. Lower information criterion indicates better posterior predictive performance, and therefore a better fit to the experiments (dashed red ellipse).

Appendix Fig. S8

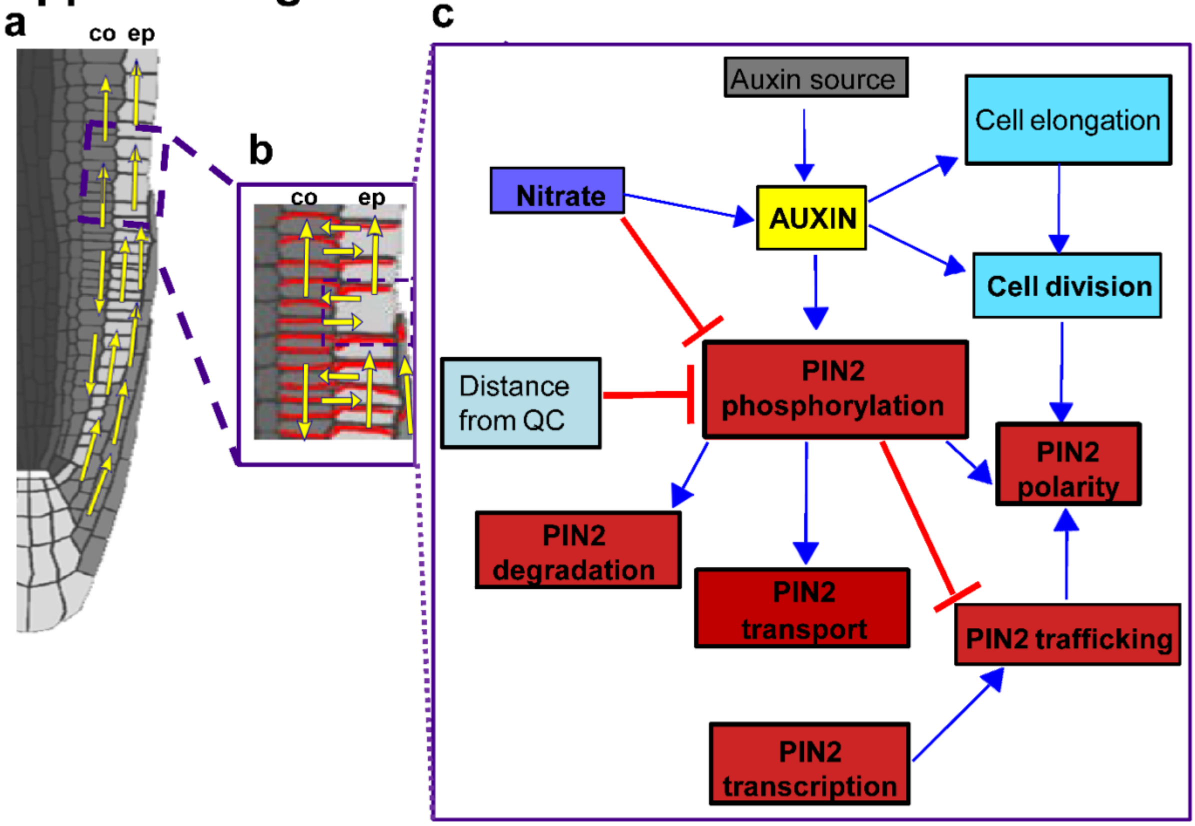

Appendix Fig. S8 | Model diagram.

a. Schematic cross-section of the root of Arabidopsis thaliana, showing the auxin flow (yellow arrows) in epidermis (ep) and cortex (co). **b.** Detailed scheme of the transition zone displaying lateral exchange of auxin (yellow arrows) between epidermis (ep) and cortex (co) achieved by PIN2 lateralization (red). **c.** The graphical chart represents the interactions between molecular and structural components assumed in the computational model. Blue lines represent positive interactions whereas red lines denote inhibitory effects, based on published data and our experimental results. The two main regulators considered in the model are auxin and nitrate levels, which exert antithetic effect on PIN2 dynamics through phosphorylation.

A.

ammonium

1.
```
Position = 1, Treatment = ammonium:
 Tissue    emmean       SE  df asymp.LCL asymp.UCL .group
 cortex    0.0362 0.002373 Inf   0.03085    0.0415  a
 epidermis 0.0416 0.002734 Inf   0.03554    0.0478  a
```
2.
```
Position = 2, Treatment = ammonium:
 Tissue    emmean       SE  df asymp.LCL asymp.UCL .group
 cortex    0.0388 0.002548 Inf   0.03312    0.0445  a
 epidermis 0.0369 0.002419 Inf   0.03145    0.0423  a
```
3.
```
Position = 3, Treatment = ammonium:
 Tissue    emmean       SE  df asymp.LCL asymp.UCL .group
 cortex    0.0354 0.002326 Inf   0.03023    0.0406  a
 epidermis 0.0378 0.002480 Inf   0.03224    0.0433  a
```
4.
```
Position = 4, Treatment = ammonium:
 Tissue    emmean       SE  df asymp.LCL asymp.UCL .group
 cortex    0.0390 0.002557 Inf   0.03324    0.0447  a
 epidermis 0.0369 0.002423 Inf   0.03150    0.0423  a
```
5.
```
Position = 5, Treatment = ammonium:
 Tissue    emmean       SE  df asymp.LCL asymp.UCL .group
 cortex    0.0387 0.002539 Inf   0.03301    0.0444  a
 epidermis 0.0366 0.002403 Inf   0.03124    0.0420  a
```
6.
```
Position = 6, Treatment = ammonium:
 Tissue    emmean       SE  df asymp.LCL asymp.UCL .group
 cortex    0.0398 0.002610 Inf   0.03393    0.0456  a
 epidermis 0.0415 0.002725 Inf   0.03542    0.0476  a
```
7.
```
Position = 7, Treatment = ammonium:
 Tissue    emmean       SE  df asymp.LCL asymp.UCL .group
 cortex    0.0444 0.002916 Inf   0.03791    0.0509  a
 epidermis 0.0434 0.002850 Inf   0.03705    0.0498  a
```
8.
```
Position = 8, Treatment = ammonium:
 Tissue    emmean       SE  df asymp.LCL asymp.UCL .group
 cortex    0.0426 0.002798 Inf   0.03637    0.0489  a
 epidermis 0.0408 0.002676 Inf   0.03479    0.0468  a
```
9.
```
Position = 9, Treatment = ammonium:
 Tissue    emmean       SE  df asymp.LCL asymp.UCL .group
 cortex    0.0426 0.002795 Inf   0.03634    0.0488  a
 epidermis 0.0392 0.002570 Inf   0.03342    0.0449  a
```
10.
```
Position = 10, Treatment = ammonium:
 Tissue    emmean       SE  df asymp.LCL asymp.UCL .group
 cortex    0.0448 0.002942 Inf   0.03824    0.0514  a
 epidermis 0.0380 0.002497 Inf   0.03246    0.0436  a
```
11.
```
Position = 11, Treatment = ammonium:
 Tissue    emmean       SE  df asymp.LCL asymp.UCL .group
 cortex    0.0472 0.003096 Inf   0.04025    0.0541  a
 epidermis 0.0341 0.002236 Inf   0.02907    0.0391   b
```
12.
```
Position = 12, Treatment = ammonium:
 Tissue    emmean       SE  df asymp.LCL asymp.UCL .group
 cortex    0.0448 0.002942 Inf   0.03825    0.0514  a
 epidermis 0.0349 0.002288 Inf   0.02974    0.0400   b
```
13.
```
Position = 13, Treatment = ammonium:
 Tissue    emmean       SE  df asymp.LCL asymp.UCL .group
 cortex    0.0426 0.002798 Inf   0.03638    0.0489  a
 epidermis 0.0352 0.002309 Inf   0.03002    0.0404   b
```
14.
```
Position = 14, Treatment = ammonium:
 Tissue    emmean       SE  df asymp.LCL asymp.UCL .group
 cortex    0.0437 0.002867 Inf   0.03727    0.0501  a
 epidermis 0.0310 0.002035 Inf   0.02646    0.0356   b
```
15.
```
Position = 15, Treatment = ammonium:
 Tissue    emmean       SE  df asymp.LCL asymp.UCL .group
 cortex    0.0403 0.002647 Inf   0.03441    0.0462  a
 epidermis 0.0277 0.001820 Inf   0.02366    0.0318   b
```
16.
```
Position = 16, Treatment = ammonium:
 Tissue    emmean       SE  df asymp.LCL asymp.UCL .group
 cortex    0.0370 0.002427 Inf   0.03156    0.0424  a
 epidermis 0.0233 0.001530 Inf   0.01989    0.0267   b
```
17.
```
Position = 17, Treatment = ammonium:
 Tissue    emmean       SE  df asymp.LCL asymp.UCL .group
 cortex    0.0330 0.002168 Inf   0.02818    0.0379  a
 epidermis 0.0212 0.001390 Inf   0.01806    0.0243   b
```
18.
```
Position = 18, Treatment = ammonium:
 Tissue    emmean       SE  df asymp.LCL asymp.UCL .group
 cortex    0.0310 0.002033 Inf   0.02643    0.0355  a
 epidermis 0.0176 0.001157 Inf   0.01504    0.0202   b
```
19.
```
Position = 19, Treatment = ammonium:
 Tissue    emmean       SE  df asymp.LCL asymp.UCL .group
 cortex    0.0298 0.002075 Inf   0.02517    0.0345  a
 epidermis 0.0127 0.000944 Inf   0.01057    0.0148   b
```
20.
```
Position = 20, Treatment = ammonium:
 Tissue    emmean       SE  df asymp.LCL asymp.UCL .group
 cortex    0.0278 0.002066 Inf   0.02314    0.0324  a
 epidermis 0.0104 0.000870 Inf   0.00842    0.0123   b
```

nitrate

```
Position = 1, Treatment = nitrate:
 Tissue    emmean       SE  df asymp.LCL asymp.UCL .group
 cortex    0.0342 0.002242 Inf   0.02914    0.0392  a
 epidermis 0.0345 0.002265 Inf   0.02944    0.0396  a

Position = 2, Treatment = nitrate:
 Tissue    emmean       SE  df asymp.LCL asymp.UCL .group
 cortex    0.0377 0.002475 Inf   0.03217    0.0432  a
 epidermis 0.0371 0.002437 Inf   0.03168    0.0426  a

Position = 3, Treatment = nitrate:
 Tissue    emmean       SE  df asymp.LCL asymp.UCL .group
 cortex    0.0376 0.002470 Inf   0.03211    0.0432  a
 epidermis 0.0367 0.002408 Inf   0.03131    0.0421  a

Position = 4, Treatment = nitrate:
 Tissue    emmean       SE  df asymp.LCL asymp.UCL .group
 cortex    0.0381 0.002498 Inf   0.03248    0.0437  a
 epidermis 0.0383 0.002511 Inf   0.03264    0.0439  a

Position = 5, Treatment = nitrate:
 Tissue    emmean       SE  df asymp.LCL asymp.UCL .group
 cortex    0.0425 0.002790 Inf   0.03627    0.0487  a
 epidermis 0.0391 0.002567 Inf   0.03337    0.0448  a

Position = 6, Treatment = nitrate:
 Tissue    emmean       SE  df asymp.LCL asymp.UCL .group
 cortex    0.0414 0.002720 Inf   0.03536    0.0475  a
 epidermis 0.0366 0.002401 Inf   0.03122    0.0420  a

Position = 7, Treatment = nitrate:
 Tissue    emmean       SE  df asymp.LCL asymp.UCL .group
 cortex    0.0406 0.002666 Inf   0.03466    0.0466  a
 epidermis 0.0364 0.002392 Inf   0.03110    0.0418  a

Position = 8, Treatment = nitrate:
 Tissue    emmean       SE  df asymp.LCL asymp.UCL .group
 cortex    0.0397 0.002606 Inf   0.03387    0.0455  a
 epidermis 0.0369 0.002424 Inf   0.03152    0.0424  a

Position = 9, Treatment = nitrate:
 Tissue    emmean       SE  df asymp.LCL asymp.UCL .group
 cortex    0.0423 0.002776 Inf   0.03609    0.0485  a
 epidermis 0.0392 0.002576 Inf   0.03349    0.0450  a

Position = 10, Treatment = nitrate:
 Tissue    emmean       SE  df asymp.LCL asymp.UCL .group
 cortex    0.0431 0.002831 Inf   0.03680    0.0495  a
 epidermis 0.0394 0.002583 Inf   0.03359    0.0451  a

Position = 11, Treatment = nitrate:
 Tissue    emmean       SE  df asymp.LCL asymp.UCL .group
 cortex    0.0408 0.002677 Inf   0.03480    0.0468  a
 epidermis 0.0370 0.002432 Inf   0.03161    0.0425  a

Position = 12, Treatment = nitrate:
 Tissue    emmean       SE  df asymp.LCL asymp.UCL .group
 cortex    0.0424 0.002783 Inf   0.03618    0.0486  a
 epidermis 0.0354 0.002326 Inf   0.03024    0.0406  a

Position = 13, Treatment = nitrate:
 Tissue    emmean       SE  df asymp.LCL asymp.UCL .group
 cortex    0.0410 0.002688 Inf   0.03494    0.0470  a
 epidermis 0.0332 0.002182 Inf   0.02836    0.0381   b

Position = 14, Treatment = nitrate:
 Tissue    emmean       SE  df asymp.LCL asymp.UCL .group
 cortex    0.0420 0.002756 Inf   0.03582    0.0481  a
 epidermis 0.0313 0.002056 Inf   0.02673    0.0359   b

Position = 15, Treatment = nitrate:
 Tissue    emmean       SE  df asymp.LCL asymp.UCL .group
 cortex    0.0421 0.002761 Inf   0.03590    0.0482  a
 epidermis 0.0302 0.001982 Inf   0.02576    0.0346   b

Position = 16, Treatment = nitrate:
 Tissue    emmean       SE  df asymp.LCL asymp.UCL .group
 cortex    0.0394 0.002588 Inf   0.03365    0.0452  a
 epidermis 0.0252 0.001652 Inf   0.02148    0.0289   b

Position = 17, Treatment = nitrate:
 Tissue    emmean       SE  df asymp.LCL asymp.UCL .group
 cortex    0.0373 0.002448 Inf   0.03182    0.0428  a
 epidermis 0.0256 0.001731 Inf   0.02176    0.0295   b

Position = 18, Treatment = nitrate:
 Tissue    emmean       SE  df asymp.LCL asymp.UCL .group
 cortex    0.0332 0.002179 Inf   0.02832    0.0381  a
 epidermis 0.0241 0.001676 Inf   0.02032    0.0278   b

Position = 19, Treatment = nitrate:
 Tissue    emmean       SE  df asymp.LCL asymp.UCL .group
 cortex    0.0293 0.001921 Inf   0.02497    0.0336  a
 epidermis 0.0253 0.001954 Inf   0.02094    0.0297  a

Position = 20, Treatment = nitrate:
 Tissue    emmean       SE  df asymp.LCL asymp.UCL .group
 cortex    0.0254 0.001669 Inf   0.02170    0.0292  a
 epidermis 0.0218 0.001685 Inf   0.01805    0.0256  a
```

B.

epidermis

1.
```
Position = 1, Tissue = epidermis:
 Treatment emmean       SE  df asymp.LCL asymp.UCL .group
 ammonium  0.0416 0.002734 Inf   0.03554    0.0478  a
 nitrate   0.0345 0.002265 Inf   0.02944    0.0396   b
```

2.
```
Position = 2, Tissue = epidermis:
 Treatment emmean       SE  df asymp.LCL asymp.UCL .group
 ammonium  0.0369 0.002419 Inf   0.03145    0.0423  a
 nitrate   0.0371 0.002437 Inf   0.03168    0.0426  a
```

3.
```
Position = 3, Tissue = epidermis:
 Treatment emmean       SE  df asymp.LCL asymp.UCL .group
 ammonium  0.0378 0.002480 Inf   0.03224    0.0433  a
 nitrate   0.0367 0.002408 Inf   0.03131    0.0421  a
```

4.
```
Position = 4, Tissue = epidermis:
 Treatment emmean       SE  df asymp.LCL asymp.UCL .group
 ammonium  0.0369 0.002423 Inf   0.03150    0.0423  a
 nitrate   0.0383 0.002511 Inf   0.03264    0.0439  a
```

5.
```
Position = 5, Tissue = epidermis:
 Treatment emmean       SE  df asymp.LCL asymp.UCL .group
 ammonium  0.0366 0.002403 Inf   0.03124    0.0420  a
 nitrate   0.0391 0.002567 Inf   0.03337    0.0448  a
```

6.
```
Position = 6, Tissue = epidermis:
 Treatment emmean       SE  df asymp.LCL asymp.UCL .group
 ammonium  0.0415 0.002725 Inf   0.03542    0.0476  a
 nitrate   0.0366 0.002401 Inf   0.03122    0.0420  a
```

7.
```
Position = 7, Tissue = epidermis:
 Treatment emmean       SE  df asymp.LCL asymp.UCL .group
 ammonium  0.0434 0.002850 Inf   0.03705    0.0498  a
 nitrate   0.0364 0.002392 Inf   0.03110    0.0418  a
```

8.
```
Position = 8, Tissue = epidermis:
 Treatment emmean       SE  df asymp.LCL asymp.UCL .group
 ammonium  0.0408 0.002676 Inf   0.03479    0.0468  a
 nitrate   0.0369 0.002424 Inf   0.03152    0.0424  a
```

9.
```
Position = 9, Tissue = epidermis:
 Treatment emmean       SE  df asymp.LCL asymp.UCL .group
 ammonium  0.0392 0.002570 Inf   0.03342    0.0449  a
 nitrate   0.0392 0.002576 Inf   0.03349    0.0450  a
```

10.
```
Position = 10, Tissue = epidermis:
 Treatment emmean       SE  df asymp.LCL asymp.UCL .group
 ammonium  0.0380 0.002497 Inf   0.03246    0.0436  a
 nitrate   0.0394 0.002583 Inf   0.03359    0.0451  a
```

11.
```
Position = 11, Tissue = epidermis:
 Treatment emmean       SE  df asymp.LCL asymp.UCL .group
 ammonium  0.0341 0.002236 Inf   0.02907    0.0391  a
 nitrate   0.0370 0.002432 Inf   0.03161    0.0425  a
```

12.
```
Position = 12, Tissue = epidermis:
 Treatment emmean       SE  df asymp.LCL asymp.UCL .group
 ammonium  0.0349 0.002288 Inf   0.02974    0.0400  a
 nitrate   0.0354 0.002326 Inf   0.03024    0.0406  a
```

13.
```
Position = 13, Tissue = epidermis:
 Treatment emmean       SE  df asymp.LCL asymp.UCL .group
 ammonium  0.0352 0.002309 Inf   0.03002    0.0404  a
 nitrate   0.0332 0.002182 Inf   0.02836    0.0381  a
```

14.
```
Position = 14, Tissue = epidermis:
 Treatment emmean       SE  df asymp.LCL asymp.UCL .group
 ammonium  0.0310 0.002035 Inf   0.02646    0.0356  a
 nitrate   0.0313 0.002056 Inf   0.02673    0.0359  a
```

15.
```
Position = 15, Tissue = epidermis:
 Treatment emmean       SE  df asymp.LCL asymp.UCL .group
 ammonium  0.0277 0.001820 Inf   0.02366    0.0318  a
 nitrate   0.0302 0.001982 Inf   0.02576    0.0346  a
```

16.
```
Position = 16, Tissue = epidermis:
 Treatment emmean       SE  df asymp.LCL asymp.UCL .group
 ammonium  0.0233 0.001530 Inf   0.01989    0.0267  a
 nitrate   0.0252 0.001652 Inf   0.02148    0.0289  a
```

17.
```
Position = 17, Tissue = epidermis:
 Treatment emmean       SE  df asymp.LCL asymp.UCL .group
 ammonium  0.0212 0.001390 Inf   0.01806    0.0243  a
 nitrate   0.0256 0.001731 Inf   0.02176    0.0295   b
```

18.
```
Position = 18, Tissue = epidermis:
 Treatment emmean       SE  df asymp.LCL asymp.UCL .group
 ammonium  0.0176 0.001157 Inf   0.01504    0.0202  a
 nitrate   0.0241 0.001676 Inf   0.02032    0.0278   b
```

19.
```
Position = 19, Tissue = epidermis:
 Treatment emmean       SE  df asymp.LCL asymp.UCL .group
 ammonium  0.0127 0.000944 Inf   0.01057    0.0148  a
 nitrate   0.0253 0.001954 Inf   0.02094    0.0297   b
```

20.
```
Position = 20, Tissue = epidermis:
 Treatment emmean       SE  df asymp.LCL asymp.UCL .group
 ammonium  0.0104 0.000870 Inf   0.00842    0.0123  a
 nitrate   0.0218 0.001685 Inf   0.01805    0.0256   b
```

cortex

```
Position = 1, Tissue = cortex:
 Treatment emmean       SE  df asymp.LCL asymp.UCL .group
 ammonium  0.0362 0.002373 Inf   0.03085    0.0415  a
 nitrate   0.0342 0.002242 Inf   0.02914    0.0392  a
```

```
Position = 2, Tissue = cortex:
 Treatment emmean       SE  df asymp.LCL asymp.UCL .group
 ammonium  0.0388 0.002548 Inf   0.03312    0.0445  a
 nitrate   0.0377 0.002475 Inf   0.03217    0.0432  a
```

```
Position = 3, Tissue = cortex:
 Treatment emmean       SE  df asymp.LCL asymp.UCL .group
 ammonium  0.0354 0.002326 Inf   0.03023    0.0406  a
 nitrate   0.0376 0.002470 Inf   0.03211    0.0432  a
```

```
Position = 4, Tissue = cortex:
 Treatment emmean       SE  df asymp.LCL asymp.UCL .group
 ammonium  0.0390 0.002557 Inf   0.03324    0.0447  a
 nitrate   0.0381 0.002498 Inf   0.03248    0.0437  a
```

```
Position = 5, Tissue = cortex:
 Treatment emmean       SE  df asymp.LCL asymp.UCL .group
 ammonium  0.0387 0.002539 Inf   0.03301    0.0444  a
 nitrate   0.0425 0.002790 Inf   0.03627    0.0487  a
```

```
Position = 6, Tissue = cortex:
 Treatment emmean       SE  df asymp.LCL asymp.UCL .group
 ammonium  0.0398 0.002610 Inf   0.03393    0.0456  a
 nitrate   0.0414 0.002720 Inf   0.03536    0.0475  a
```

```
Position = 7, Tissue = cortex:
 Treatment emmean       SE  df asymp.LCL asymp.UCL .group
 ammonium  0.0444 0.002916 Inf   0.03791    0.0509  a
 nitrate   0.0406 0.002666 Inf   0.03466    0.0466  a
```

```
Position = 8, Tissue = cortex:
 Treatment emmean       SE  df asymp.LCL asymp.UCL .group
 ammonium  0.0426 0.002798 Inf   0.03637    0.0489  a
 nitrate   0.0397 0.002606 Inf   0.03387    0.0455  a
```

```
Position = 9, Tissue = cortex:
 Treatment emmean       SE  df asymp.LCL asymp.UCL .group
 ammonium  0.0426 0.002795 Inf   0.03634    0.0488  a
 nitrate   0.0423 0.002776 Inf   0.03609    0.0485  a
```

```
Position = 10, Tissue = cortex:
 Treatment emmean       SE  df asymp.LCL asymp.UCL .group
 ammonium  0.0448 0.002942 Inf   0.03824    0.0514  a
 nitrate   0.0431 0.002831 Inf   0.03680    0.0495  a
```

```
Position = 11, Tissue = cortex:
 Treatment emmean       SE  df asymp.LCL asymp.UCL .group
 ammonium  0.0472 0.003096 Inf   0.04025    0.0541  a
 nitrate   0.0408 0.002677 Inf   0.03480    0.0468  a
```

```
Position = 12, Tissue = cortex:
 Treatment emmean       SE  df asymp.LCL asymp.UCL .group
 ammonium  0.0448 0.002942 Inf   0.03825    0.0514  a
 nitrate   0.0424 0.002783 Inf   0.03618    0.0486  a
```

```
Position = 13, Tissue = cortex:
 Treatment emmean       SE  df asymp.LCL asymp.UCL .group
 ammonium  0.0426 0.002798 Inf   0.03638    0.0489  a
 nitrate   0.0410 0.002688 Inf   0.03494    0.0470  a
```

```
Position = 14, Tissue = cortex:
 Treatment emmean       SE  df asymp.LCL asymp.UCL .group
 ammonium  0.0437 0.002867 Inf   0.03727    0.0501  a
 nitrate   0.0420 0.002756 Inf   0.03582    0.0481  a
```

```
Position = 15, Tissue = cortex:
 Treatment emmean       SE  df asymp.LCL asymp.UCL .group
 ammonium  0.0403 0.002647 Inf   0.03441    0.0462  a
 nitrate   0.0421 0.002761 Inf   0.03590    0.0482  a
```

```
Position = 16, Tissue = cortex:
 Treatment emmean       SE  df asymp.LCL asymp.UCL .group
 ammonium  0.0370 0.002427 Inf   0.03156    0.0424  a
 nitrate   0.0394 0.002588 Inf   0.03365    0.0452  a
```

```
Position = 17, Tissue = cortex:
 Treatment emmean       SE  df asymp.LCL asymp.UCL .group
 ammonium  0.0330 0.002168 Inf   0.02818    0.0379  a
 nitrate   0.0373 0.002448 Inf   0.03182    0.0428  a
```

```
Position = 18, Tissue = cortex:
 Treatment emmean       SE  df asymp.LCL asymp.UCL .group
 ammonium  0.0310 0.002033 Inf   0.02643    0.0355  a
 nitrate   0.0332 0.002179 Inf   0.02832    0.0381  a
```

```
Position = 19, Tissue = cortex:
 Treatment emmean       SE  df asymp.LCL asymp.UCL .group
 ammonium  0.0298 0.002075 Inf   0.02517    0.0345  a
 nitrate   0.0293 0.001921 Inf   0.02497    0.0336  a
```

```
Position = 20, Tissue = cortex:
 Treatment emmean       SE  df asymp.LCL asymp.UCL .group
 ammonium  0.0278 0.002066 Inf   0.02314    0.0324  a
 nitrate   0.0254 0.001669 Inf   0.02170    0.0292  a
```

c.

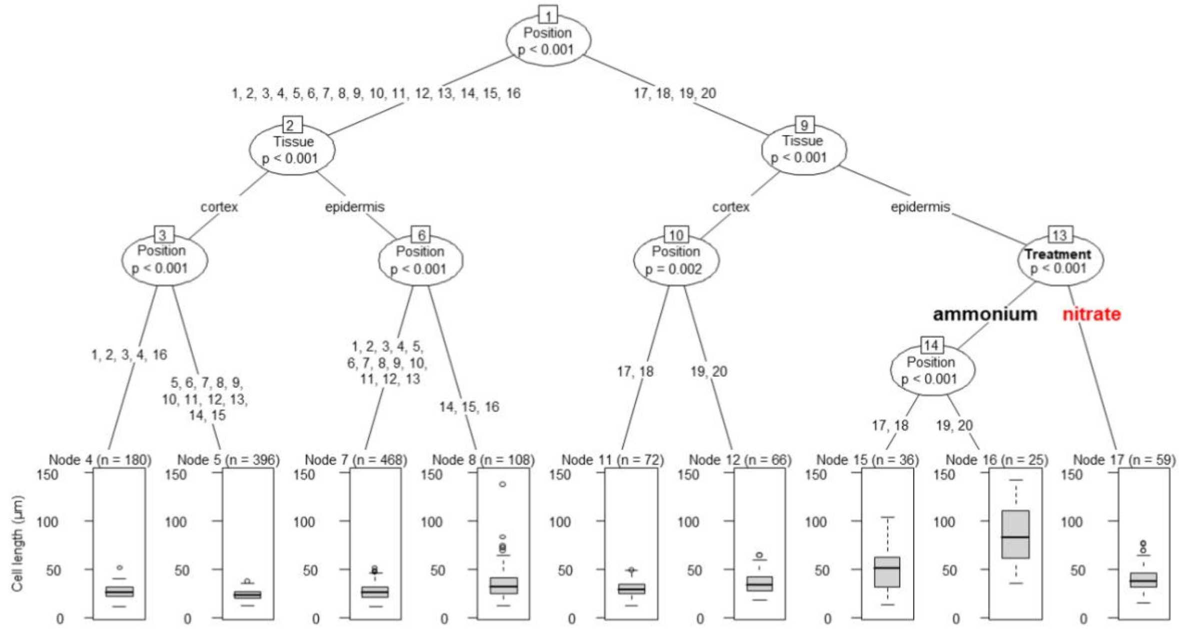

Appendix Fig. S9 | Statistical reasoning.

a. Estimated marginal mean (EMM) comparisons of cell lengths in different tissues (epidermis vs cortex) at each cell position (1-20 from QC) for each treatment (ammonium vs nitrate) applied on a generalized linear model (GLM). Significant differences (p<0.01) are highlighted in red. **b.** EMM comparisons of cell lengths in different treatments (ammonium vs nitrate) at each cell position (1-20 from QC) for epidermis vs cortex on the same GLM model. Significant differences (p<0.01) are highlighted in red. **c.** A decision tree based on recursive partitioning analysis shows the hierarchical importance of each treatment, tissue and cell position variable on cell length differences.
