# Supplementary material for: Modulation of plant root growth by nitrogen source‐defined regulation of polar auxin transport
Source: EMBO J. 2021 Jan 5;40(3):e106862. doi: 10.15252/embj.2020106862 (PMC7849315; doi:10.15252/embj.2020106862)
Supplement: Supplementary file 3 — Expanded View Figures PDF [file EMBJ-40-e106862-s002.pdf]

Expanded View Figures

Figure EV1. Additional data supporting the distinct growth kinetics of Col-0 roots transferred to ammonium or nitrate supplemented media.

A Primary root length (mm) of Col-0 seedlings 6 and 12 HAT and 1, 2, and 4 days after transfer (DAT) to ammonium (black) or nitrate (red) supplemented medium. At least 34 roots were measured per time point per treatment. The statistical significance was evaluated with ANOVA at $P < 0.05$ (*) and $P < 0.001$ (***). The box chart components are defined as, box (25–75%), central band (median line), and central box (mean), and the range is within 1.5IQR.

B On the left, schematic representation of distinct root zones: Meristematic Zone (MZ), Transition Zone (TZ, which is interpolated between the apical meristem and the subapical elongation zone), and Elongation Zone (EZ). Boxes highlight the borders of the specified root zones (gray box for ammonium and red for nitrate). On the right, Col-0 epidermal cells length was measured along the root tip (from QC), grouped based on their cell length (*x* axis) and were plotted against the average cell number per group per root (*y* axis) in both conditions (ammonium, black and nitrate, red). Note the higher cell number in case of nitrate (red) in the group (30–40 μm, representing TZ). Data are derived from 3 biological replicates, and total number of analyzed roots is $n = 18$ in each case.

C Maximal cell length (measured at the end of the elongation zone) of Col-0 roots 12 HAT either to ammonium (black) or nitrate (red). 13 roots per treatment, 3 cells per root were analyzed. The box chart components are defined as, box (25–75%), central band (median line), and central box (mean), and the range is within 1.5IQR.

D, E Comparison of cell length measurements along epidermis (D) and cortex (E) upon ammonium (black and red) and nitrate (blue and green) treatments. Column bars denote the geometric mean of cell length at the respective positions. Lines represent a polynomial regression fit. Data are derived from 3 biological replicates, and total number of analyzed roots is $n = 18$ in each case.

Source data are available online for this figure.

© 2020 The Authors

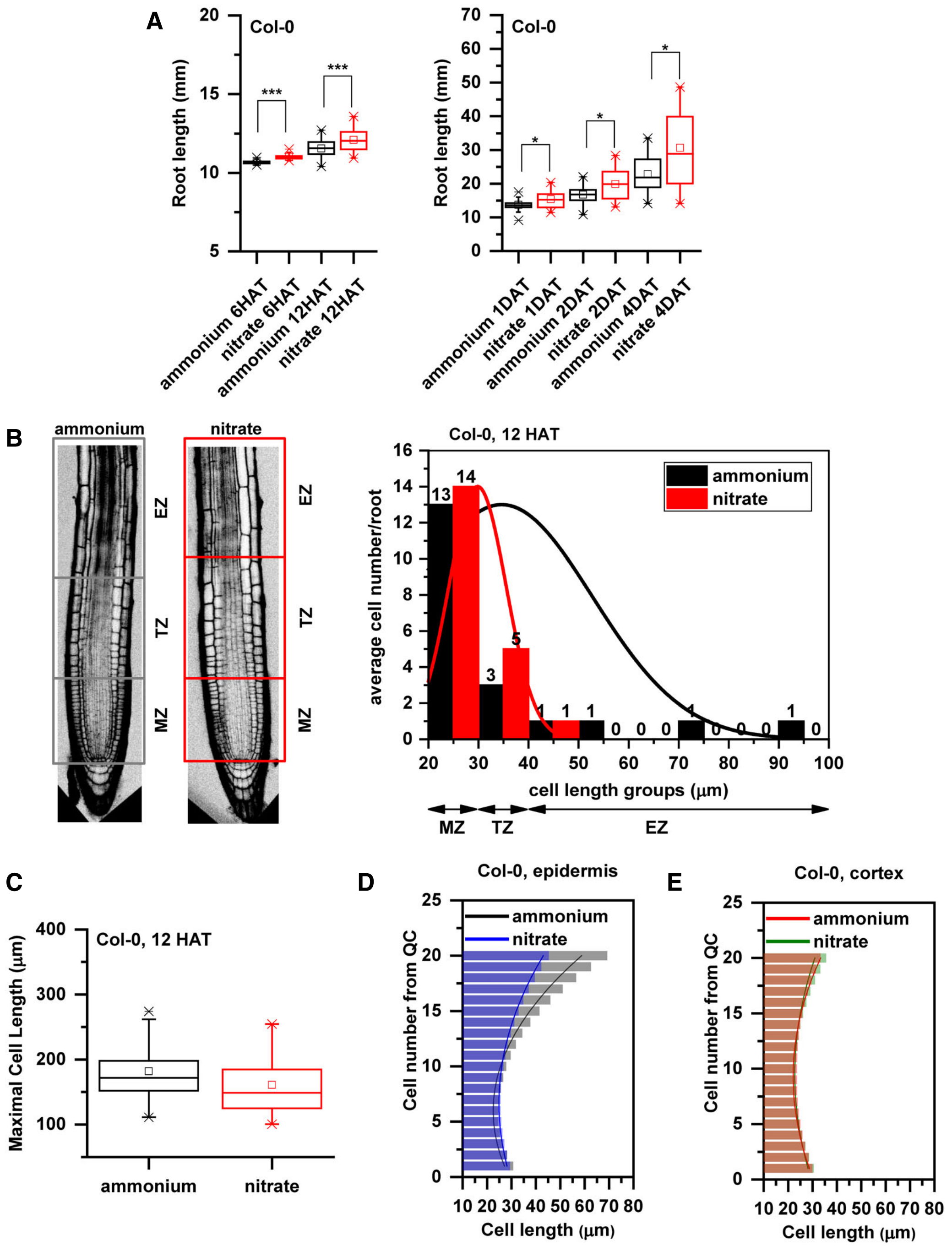
A
Col-0
Root length (mm)
ammonium 6HAT
nitrate 6HAT
ammonium 12HAT
nitrate 12HAT
ammonium 1DAT
nitrate 1DAT
ammonium 2DAT
nitrate 2DAT
ammonium 4DAT
nitrate 4DAT
B
ammonium
nitrate
EZ
TZ
MZ
Col-0, 12 HAT
average cell number/root
cell length groups (µm)
C
Col-0, 12 HAT
Maximal Cell Length (µm)
D
Col-0, epidermis
Cell number from QC
Cell length (µm)
E
Col-0, cortex

Figure EV1.

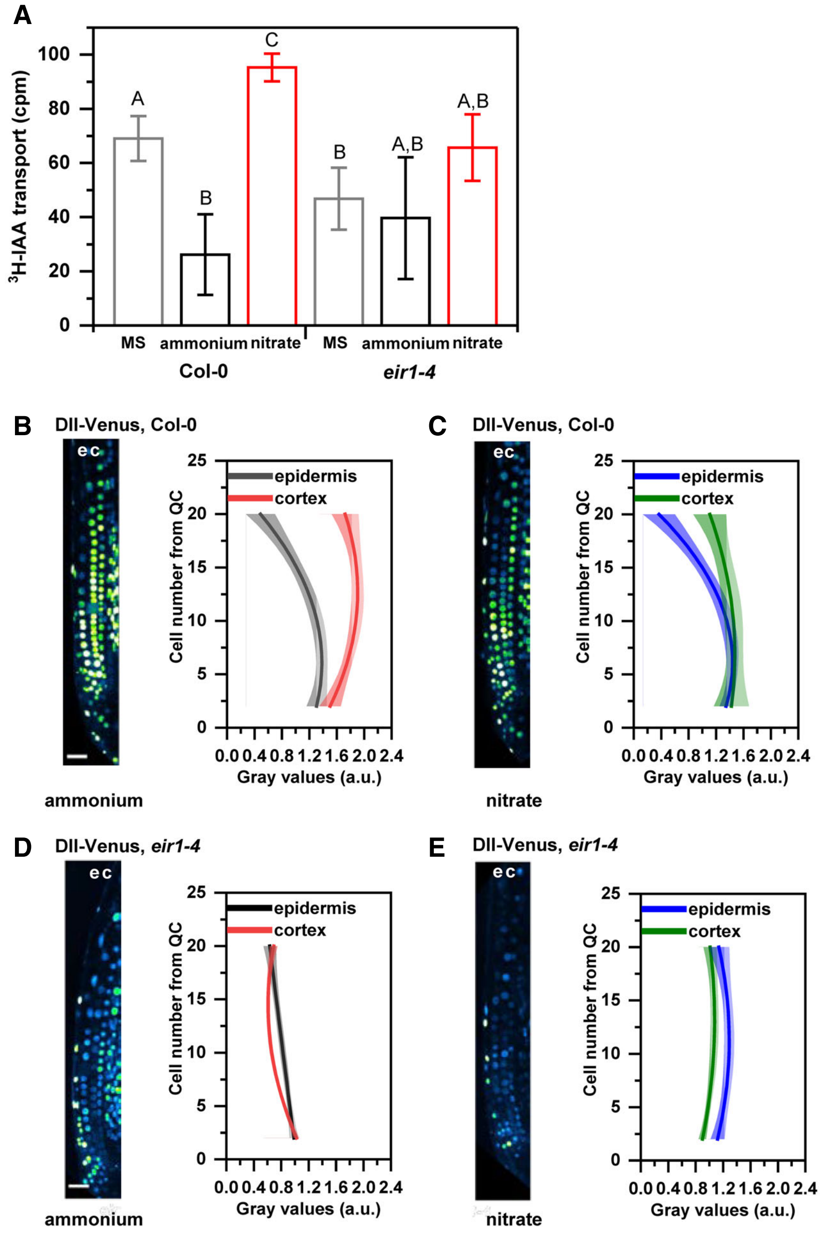

Figure EV2. Monitoring basipetal auxin transport and auxin response at root tips of Col-0 and *eir1-4* roots transferred to ammonium or nitrate-containing medium.

A Basipetal (shootward) auxin transport measurements in Col-0 and *eir1-4* roots grown on control Murashige and Skoog (MS) or with either nitrate or ammonium supplied media. ^{3}H-IAA was applied at the root tip of 7 DAG wild-type (Col-0) or *eir1-4* seedlings. Radioactivity was measured 6 h after application of ^{3}H-IAA in root segments after excision of the apical ≈ 1 mm of the root tip. Values shown are the geometric mean (± standard deviation, SD) for at least 30 seedlings. The amount of auxin transported into each root segment for Col-0 and *eir1-4* was compared by ANOVA at $P < 0.05$. cpm, counts per minute.

B–E Maximum intensity Z-stack projection images of 5 DAG old Col-0 and *eir1-4* roots expressing the *DII-Venus* auxin signaling reporter 12 HAT to ammonium (B and D) or nitrate (C and E) supplemented media. “e” and “c” mark epidermis and cortex, respectively. Scale bar = 50 μm. Graphs denote normalized relative auxin levels at the respective positions. Lines represent polynomial regression fit with 95% confidence band. Data are derived from measurements of $n = 8$ (ammonium) and $n = 10$ (nitrate) roots of Col-0 and $n = 10$ roots of *eir1-4* per condition.

Source data are available online for this figure.

© 2020 The Authors

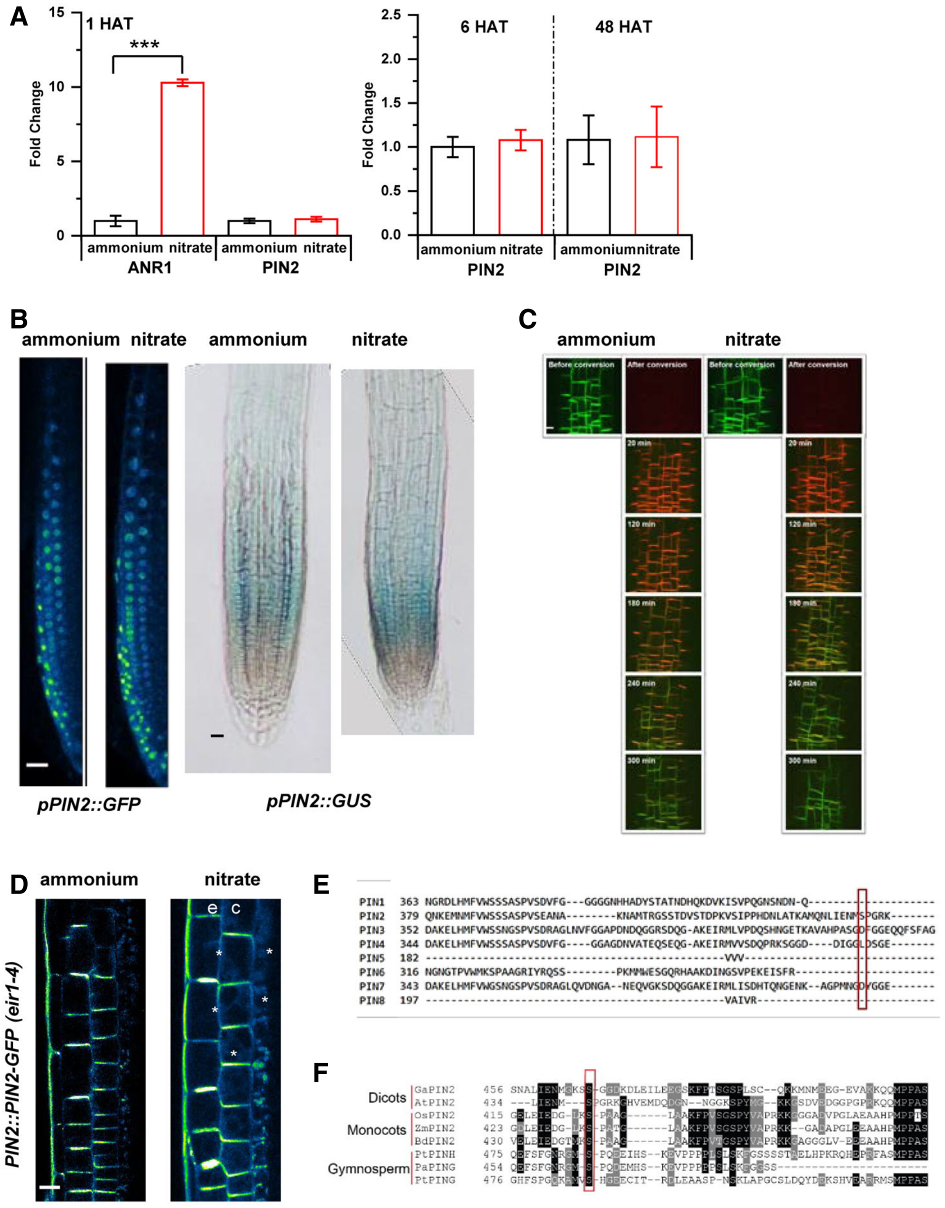

Figure EV3. Additional data supporting distinct effects of nitrogen sources on PIN2.

A RT–qPCR analysis of *PIN2* expression normalized to *UBQ10 (AT4G05320)* levels in Col-0 roots 1, 6 and 48 HAT to ammonium or nitrate. As a positive control, expression of *ANR1* (nitrate responsive MADS-box transcription factor) was quantified. All RT–qPCR reactions were carried out with biological and technical triplicates. Statistical difference was calculated with a *t*-test (*P* value ***<0.001). Error bars represent mean ± SD. The experiment was done three times.

B *PIN2* promoter activity was monitored in *pPIN2::nlsGFP* and *pPIN2::GUS* expressing roots 12 HAT to ammonium or nitrate. Scale bars = 50 μm.

C Confocal microscopic images of *PIN2::PIN2-DENDRA* fluorescence in the same area of the root transition zones 12 HAT to ammonium or nitrate before and after photoconversion (0, 20, 120, 180, 240, 300 min). Scale bar = 20 μm.

D Multiphoton microscopic image showing polarity changes of PIN2 expression upon nitrate treatment. "e" and "c" denote epidermis and cortex respectively. White arrows mark lateralization of the PIN2-GFP signal in cortex cells (c). Scale bar = 10 μm.

E Protein sequence alignment of members of the *Arabidopsis* PIN protein family. Ser439 of PIN2 and the corresponding residues of other PIN family members are marked by a red box.

F PIN2 protein sequence alignment shows evolutionary conservation of Ser439 in representative members of Gymnosperms, Monocots, and Dicots. From Gymnosperms *Picea abies* (Pa) and *Pinus taeda* (Pt) PIN2 proteins (PtPING, PtPINH, PaPING), from Monocots *Zea mays* (Zm), *Brachypodium distachyon* (Bd), and *Oryza sativa* (Os) PIN2 proteins (ZmPIN2, BdPIN2, OsPIN2) and from Dicots *Gossypium arboreun* (Ga) and *Arabidopsis thaliana* (At) PIN2 proteins (GaPIN2, AtPIN2) were used. Protein alignments were created with the MEGAX software (Kumar *et al*, 2018).

Source data are available online for this figure.

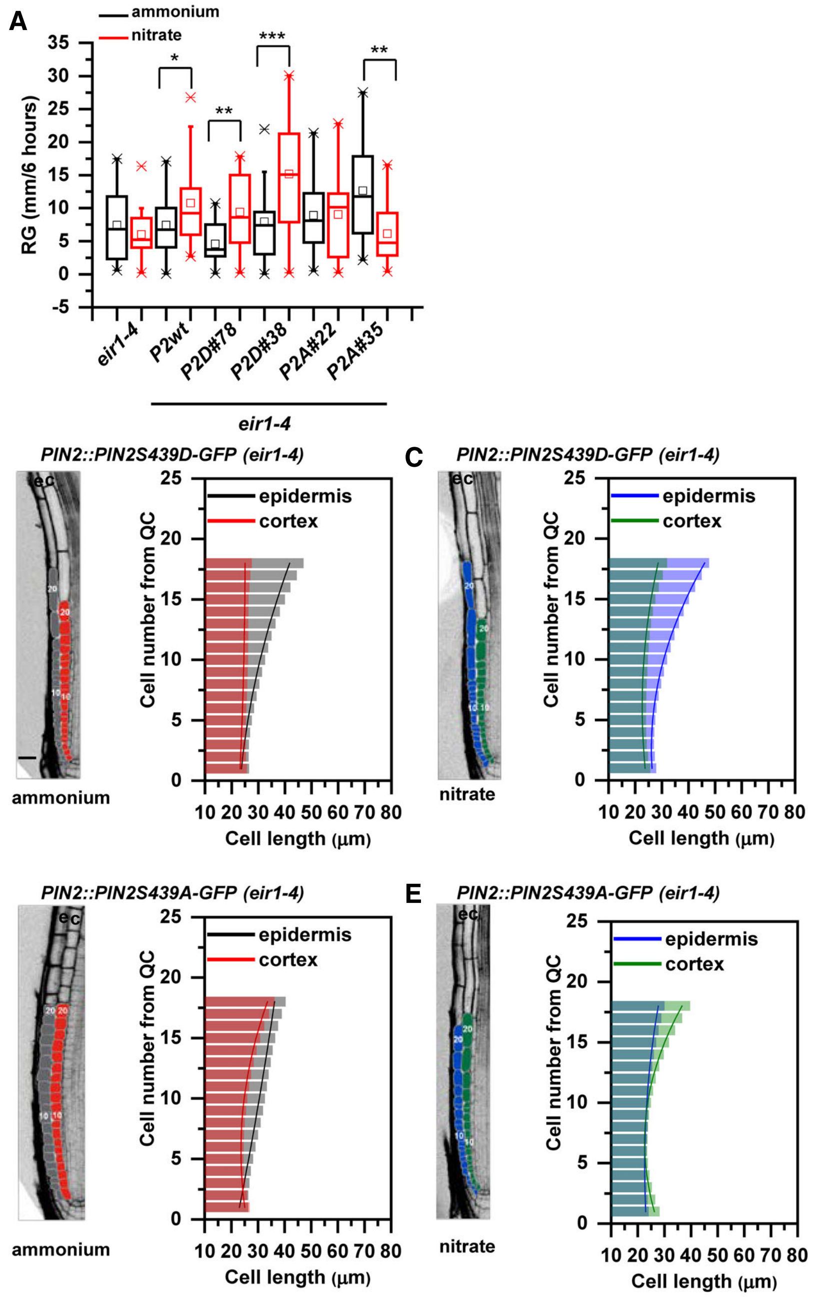

Figure EV4. Impact of PIN2S439 phosphovariants on the adaptation of the primary root growth to ammonium or nitrate provision.

A Box plot representation of root growth (µm/6 h) of *eir1-4*, Col-0, *PIN2::PIN2-GFP* (*P2wt*), two independent *PIN2::PIN2S439D-GFP* (*P2D*) lines (#78 and #38) and two independent *PIN2:PIN2S439A-GFP* (*P2A*) lines (#22 and #35) transferred to ammonium or nitrate-containing medium. At least 10 roots were analyzed per genotype per treatment. The statistical significance was evaluated with ANOVA at $P < 0.05$ (*), $P < 0.01$ (**), $P < 0.001$ (***). The box chart components are defined as, box (25–75%), central band (median line), and central box (mean), and the range is within 1.5IQR.

B–E Optical, longitudinal sections of 5 DAG old roots expressing *PIN2S439D-GFP* (B and C) and *PIN2S439A-GFP* (D and E) 12 HAT to ammonium (B and D) or nitrate (C and D) supplemented media. The first 20-20 epidermal and cortex cells (from quiescent center (QC)) are highlighted in gray and in red on ammonium (B and D) and in blue and green on nitrate (C and E), respectively. Scale bar = 30 µm. Column bars denote the geometric mean of cell length at the respective positions. Lines represent a polynomial regression fit, with calculated slopes between cells 10 and 20 of ammonium-PIN2S439D-epidermis: 1.38867 + 0.03079, ammonium-PIN2S439D-cortex: 0.05689 + 0.00497, nitrate-PIN2S439D-epidermis: 1.92749 + 0.0727, nitrate-PIN2S439D-cortex: 0.66477 + 0.03592, ammonium-PIN2S439A-epidermis: 0.7164 ± 0.00565, ammonium-PIN2S439A-cortex: 1.09064 ± 0.05609, nitrate-PIN2S439A-epidermis: 0.53796 ± 0.0249, and nitrate-PIN2S439A-cortex: 1.61118 ± 0.09541. Data are derived from 3 biological replicates; at least 5 roots were analyzed in each case.

Source data are available online for this figure.

 © 2020 The Authors

Figure EV5. The role of NRT1.1. in the root growth adaptation to differing nitrogen sources.

A Root growth (RG in μm/min) of roots of Col-0, *chl1-5*, *chl1-9*, T101A, and T101D seedlings 12 HAT to ammonium (black) or nitrate (red). Seedlings were transferred at 7 DAG. At least 10 roots/treatment/genotype were measured. The experiment was repeated 3 times. The statistical significance was evaluated with ANOVA at $P < 0.01$ (**). The box chart components are defined as, box (25–75%), central band (median line), and central box (mean), and the range is within 1.5IQR.

B Pseudo-colored, optical longitudinal sections of PIN2 immunostained (yellow signal), 5 DAG roots of *chl1-5* and *PIN2::PIN2-GFP*, *eir1*-4 expressing roots, 12 HAT to ammonium or nitrate supplemented media. "e" denotes epidermis and "c" cortex, respectively. Scale bar = 30 μm. Box plots display the distribution of the cell membrane-derived anti-PIN2 fluorescence intensity (FI) values (in arbitrary units, a.u.) on ammonium (gray, epidermis (ep) and red, cortex (co), $n = 40$ membrane) and nitrate (blue, epidermis (ep) and green, cortex (co), $n = 40$ membrane) grown roots. 4 roots were analyzed/treatment/genotype, and 10 cells per root were quantified. The statistical significance was evaluated with ANOVA at $P < 0.05$. The box chart components are defined as, box (25–75%), central band (median line), central box (mean), and the range is within 1.5IQR.

C Pseudo-colored, longitudinal Z-stacks of *PIN1::PIN1-GFP* expressing 5 DAG roots, 12 HAT to ammonium or nitrate. Scale bar = 30 μm. Box plots display the distribution of the cell membrane-derived PIN1-GFP fluorescence intensity (FI) values (in arbitrary units, a.u.) on ammonium (black) and red (nitrate). 4 roots were analyzed/treatment, and 10 cells per root were quantified. The statistical significance was evaluated with ANOVA at $P < 0.05$. The box chart components are defined as, box (25–75%), central band (median line), and central box (mean), and the range is within 1.5IQR.

D Pseudo-colored, longitudinal Z-stacks of *PIP2::PIP2-GFP* expressing 5 DAG roots, 12 HAT to ammonium or nitrate. Scale bar = 10 μm. Box plots display the distribution of the cell membrane-derived PIP2-GFP fluorescence intensity (FI) values (in arbitrary units, a.u.) on ammonium (black) and red (nitrate). 4 roots were analyzed/treatment and 10 cells per root were quantified. The statistical significance was evaluated with ANOVA at $P < 0.05$. The box chart components are defined as, box (25–75%), central band (median line), and central box (mean), and the range is within 1.5IQR.

Source data are available online for this figure.

© 2020 The Authors

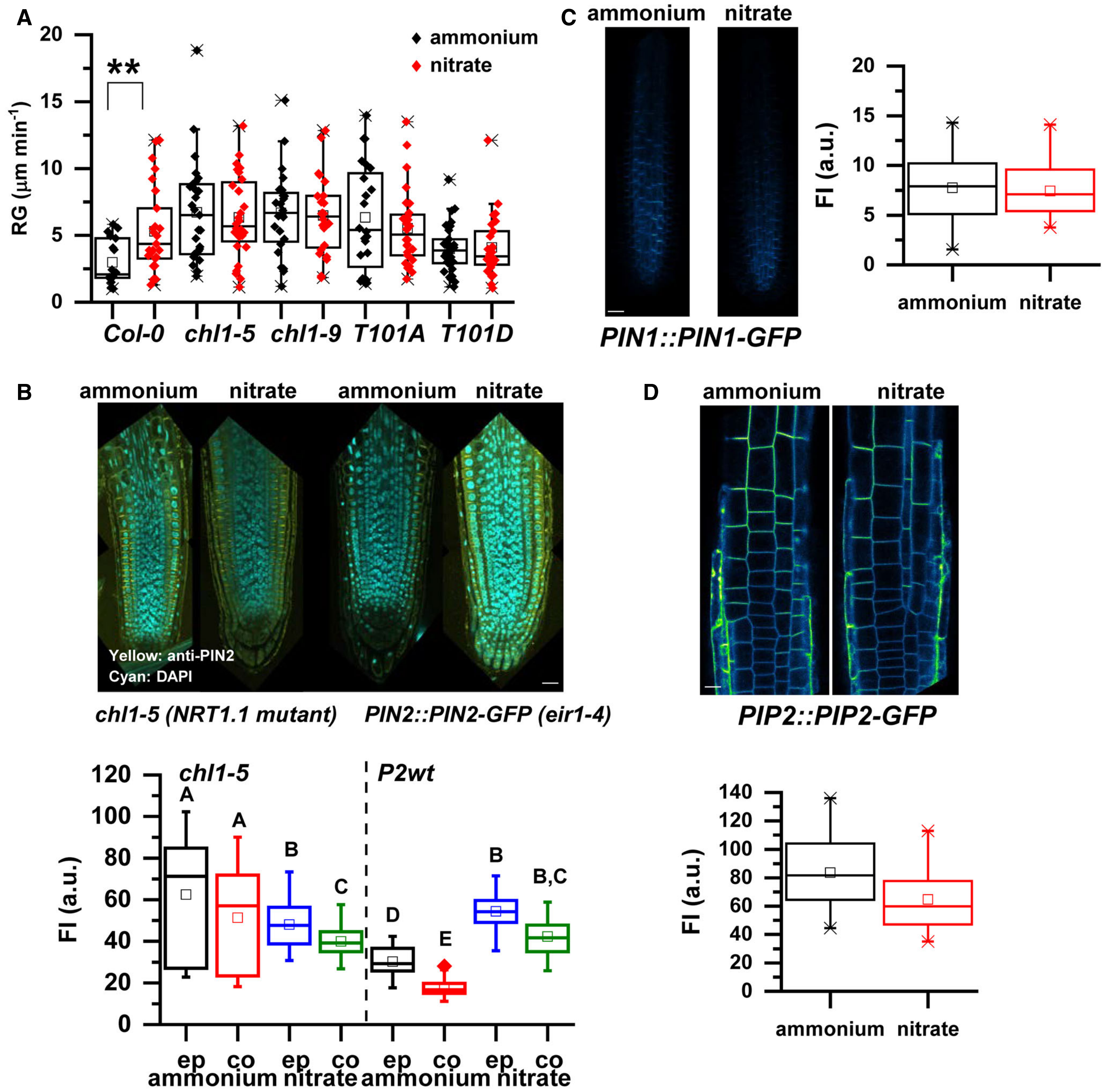

Figure EV5.

 © 2020 The Authors
