# Supplementary material for: Modulation of plant root growth by nitrogen source‐defined regulation of polar auxin transport
Source: EMBO J. 2021 Jan 5;40(3):e106862. doi: 10.15252/embj.2020106862 (PMC7849315; doi:10.15252/embj.2020106862)
Supplement: Supplementary file 5 — Movie EV2 [file EMBJ-40-e106862-s004.zip › EMBOJ-2020-106862_Movie EV2_Legends.docx]

**Movie EV2 – Related to Figure 1**

Time lapse of the transition zone of 5-day-old *Arabidopsis* roots expressing the PM marker (*WAVE131Y*) transferred to either on ammonium or nitrate amended media and imaged with a vertically oriented LSM700 microscope. Observation of roots initiated 20 minutes after transfer and images recorded every 20 minutes (9 stacks/root/ recording).
