# Supplementary material for: Modulation of plant root growth by nitrogen source‐defined regulation of polar auxin transport
Source: EMBO J. 2021 Jan 5;40(3):e106862. doi: 10.15252/embj.2020106862 (PMC7849315; doi:10.15252/embj.2020106862)
Supplement: Supplementary file 6 — Movie EV3 [file EMBJ-40-e106862-s005.zip › EMBOJ-2020-106862_Movie EV3_Legends.docx]

**Movie EV3 – Related to Figure 4c**

Z-stacks of 2-2 cells in the transition zone of 5-day-old *Arabidopsis* roots expressing *PIN2::PIN2-GFP* 12 HAT to either to ammonium or nitrate amended media and imaged with an Airyscan LSM800 microscope.
