# Supplementary material for: Modulation of plant root growth by nitrogen source‐defined regulation of polar auxin transport
Source: EMBO J. 2021 Jan 5;40(3):e106862. doi: 10.15252/embj.2020106862 (PMC7849315; doi:10.15252/embj.2020106862)
Supplement: Supplementary file 7 — Movie EV4 [file EMBJ-40-e106862-s006.zip › EMBOJ-2020-106862_Movie EV4_Legends.docx]

**Movie EV4 – Related to Figure EV3**

Time lapse of *Arabidopsis* seedlings expressing *PIN2::PIN2-GFP* (*PIN2wt*), *PIN2::PIN2S439D-GFP* (*PIN2S439D*) and *PIN2::PIN2S439A-GFP* (*PIN2S439A*). Seedlings were grown on ammonium plates for 7 days and were transferred to ammonium containing agar plates. Plates were scanned on a daily basis with an Epson Perfection V700 flatbed scanner. Images were concatenated with Fiji.
