# Supplementary material for: Modulation of plant root growth by nitrogen source‐defined regulation of polar auxin transport
Source: EMBO J. 2021 Jan 5;40(3):e106862. doi: 10.15252/embj.2020106862 (PMC7849315; doi:10.15252/embj.2020106862)
Supplement: Supplementary file 9 — Movie EV6 [file EMBJ-40-e106862-s008.zip › EMBOJ-2020-106862_Movie EV6_Legends.docx]

**Movie EV6 – Related to Figure 5a**

A simulation of static example model shows intercellular auxin transport via PIN2 auxin carrier.
