# Supplementary material for: Modulation of plant root growth by nitrogen source‐defined regulation of polar auxin transport
Source: EMBO J. 2021 Jan 5;40(3):e106862. doi: 10.15252/embj.2020106862 (PMC7849315; doi:10.15252/embj.2020106862)
Supplement: Supplementary file 11 — Movie EV8 [file EMBJ-40-e106862-s010.zip › EMBOJ-2020-106862_Movie EV8_Legends.docx]

**Movie EV8 – Related to Figure 6b**

Simulation of asynchronous growth of epidermis-cortex tissues in nitrate condition.
