# Supplementary material for: Modulation of plant root growth by nitrogen source‐defined regulation of polar auxin transport
Source: EMBO J. 2021 Jan 5;40(3):e106862. doi: 10.15252/embj.2020106862 (PMC7849315; doi:10.15252/embj.2020106862)
Supplement: Supplementary file 2 — Source Data for Expanded View and Appendix [file EMBJ-40-e106862-s016.zip › EV Figure Source Data.zip!/EV Figure Source Data/Figure EV3 Source Data.zip!/Figure EV3 Source Data/EMBOJ-2020-106862_Microscopy source data Fig EV3.pdf]

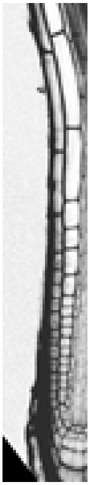

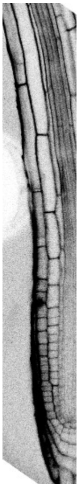

PIN2::PIN2S439D-GFP
(eir1-4)

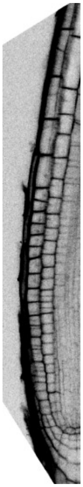

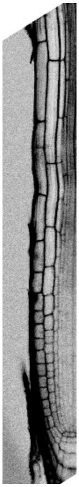

PIN2::PIN2S439A-GFP
(eir1-4)

Fig. EV3b **Fig. EV3c** **Fig. EV3d** **Fig. EV3e**
